# Characterization of a mixture of algae waste-bentonite used as adsorbent for the removal of Pb2+ from aqueous solution

**DOI:** 10.1016/j.dib.2017.12.030

**Published:** 2017-12-16

**Authors:** Eko Prasetyo Kuncoro, Thin Soedarti, Trisnadi Widyaleksono Catur Putranto, Handoko Darmokoesoemo, Nanda Rizki Abadi, Heri Septya Kusuma

**Affiliations:** a Department of Biology, Faculty of Science and Technology, Airlangga University, 60115, Indonesia; b Department of Chemistry, Faculty of Science and Technology, Airlangga University, 60115, Indonesia; c Department of Chemical Engineering, Faculty of Industrial Technology, Institut Teknologi Sepuluh Nopember, 60111, Indonesia

**Keywords:** Adsorption, Algae waste, Bentonite, Pb^2+^, Composite adsorbent

## Abstract

The usage of wastes of algae would be admirable from environmental and solid waste management point of view. Thus, herein, this data set present a facile method for providing an adsorbent from mixture of algae waste-bentonite. The prepared adsorbent was applied to remove Pb^2+^ from aqueous solution. The characterization data of the adsorbent were analyzed using FTIR and SEM-EDX methods. The FTIR test results showed that there is a shift in the wave numbers on the adsorbent that has been loaded with Pb indicating that there is an interaction between the adsorbent and Pb. The SEM-EDX test results showed that there is Pb on the adsorbent that has been loaded with Pb. It was conducted in laboratory scale and the adsorption technique was batch technique. The acquired data indicated that the adsorption of Pb^2+^ by the adsorbent prepared from mixture of algae waste-bentonite is a promising technique for treating Pb-bearing wastewaters.

**Specifications Table**

**Table:** 

Subject area	*Chemical Engineering*
More specific subject area	*Adsorption*
Type of data	*Table, image, figure*
How data was acquired	– *The uptake of Pb*^*2+*^ *by the adsorbent (q*_*e*_*) was determined based on the subtraction of the initial and final concentration of adsorbate* – *Fourier transform infrared (FTIR) spectroscopy (Shimadzu, IRPrestige 21), scanning electron microscopy with energy dispersive X-ray (SEM-EDX) spectroscopy (JEOL, JMS 5600, Tokyo, Japan) was used for determine the characteristics of the adsorbent* – *The Pb*^*2+*^ *concentration measurement was performed by Atomic Absorption Spectrophotometer (Shimadzu, AA-7000)*
Data format	*Analyzed*
Experimental factors	– *The treatment given to algae waste was drying under sunlight for several days* – *The adsorbent of algae waste-bentonite was prepared from mixture of algae waste and bentonite that have been weighed in accordance with the ratio of 1:2* – *Data of algae waste-bentonite were acquired for Pb*^*2+*^ *removal from aqueous solution*
Experimental features	*The adsorbent of algae waste-bentonite for Pb*^*2+*^*adsorption from aqueous solution*
Data source location	*Airlangga University, Surabaya, Indonesia*
Data accessibility	*Data are accessible with the article*

**Value of the data**

• The newly synthesized adsorbent has a good potential application in related of wastewater treatment.
• This data offer a simple method for preparation of adsorbent from mixture of algae waste and bentonite.
• The acquired data will be advantageous for the scientific community wanting to scale up and design an adsorption column with adsorbent of algae waste-bentonite as medium for the removal of Pb^2+^-containing waters or wastewaters.

## 1 Data

**Fig. 1:**
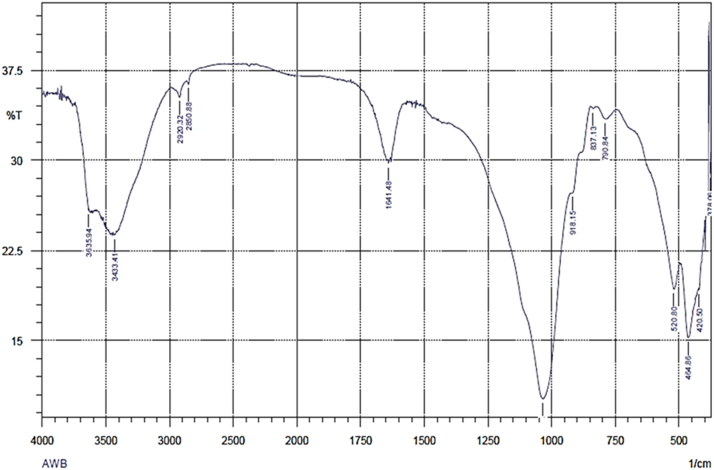
The FTIR spectrum for the adsorbent from mixture of algae waste-bentonite before adsorption.

**Fig. 2:**
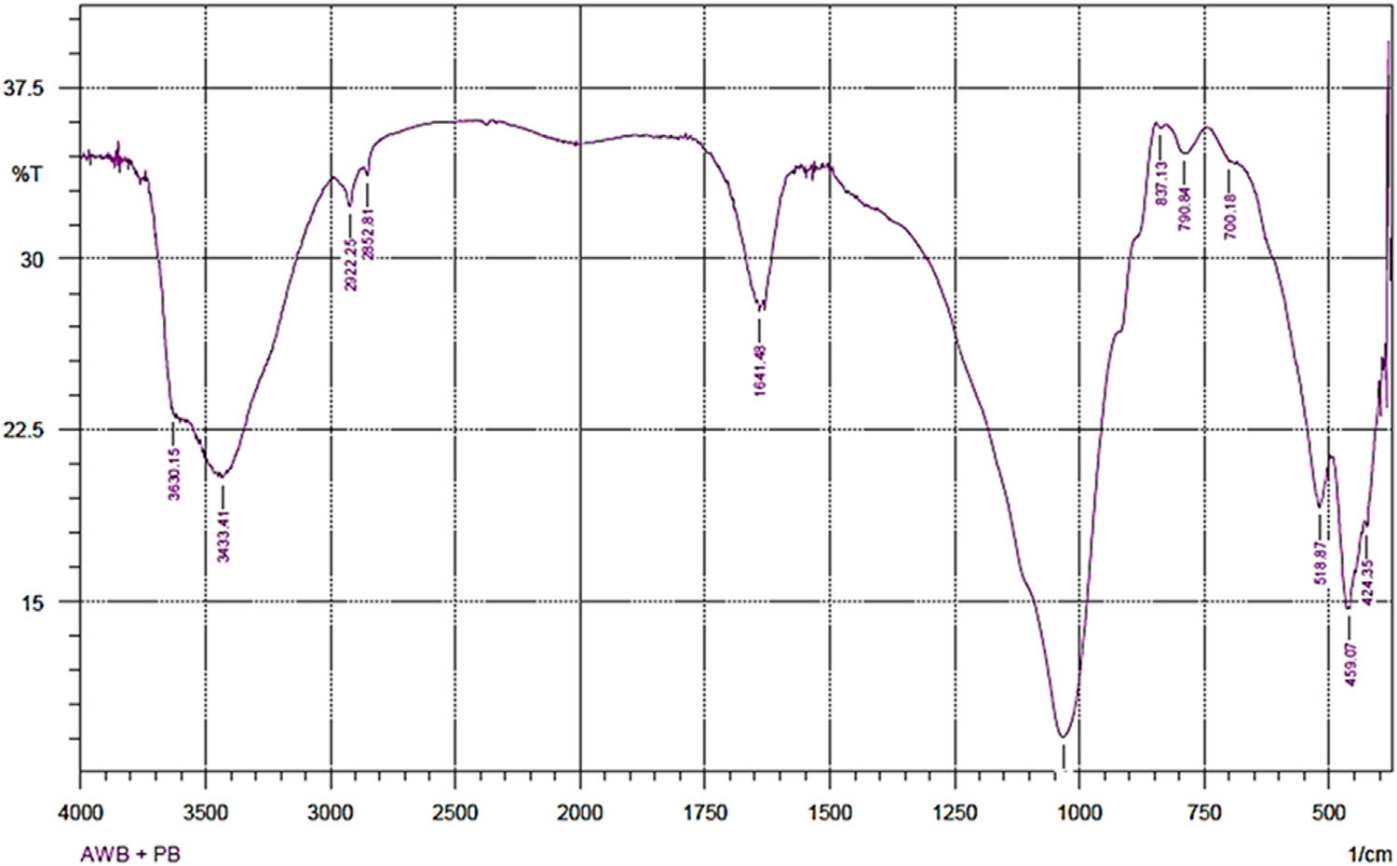
The FTIR spectrum for the adsorbent from mixture of algae waste-bentonite after adsorption.

**Fig. 3:**
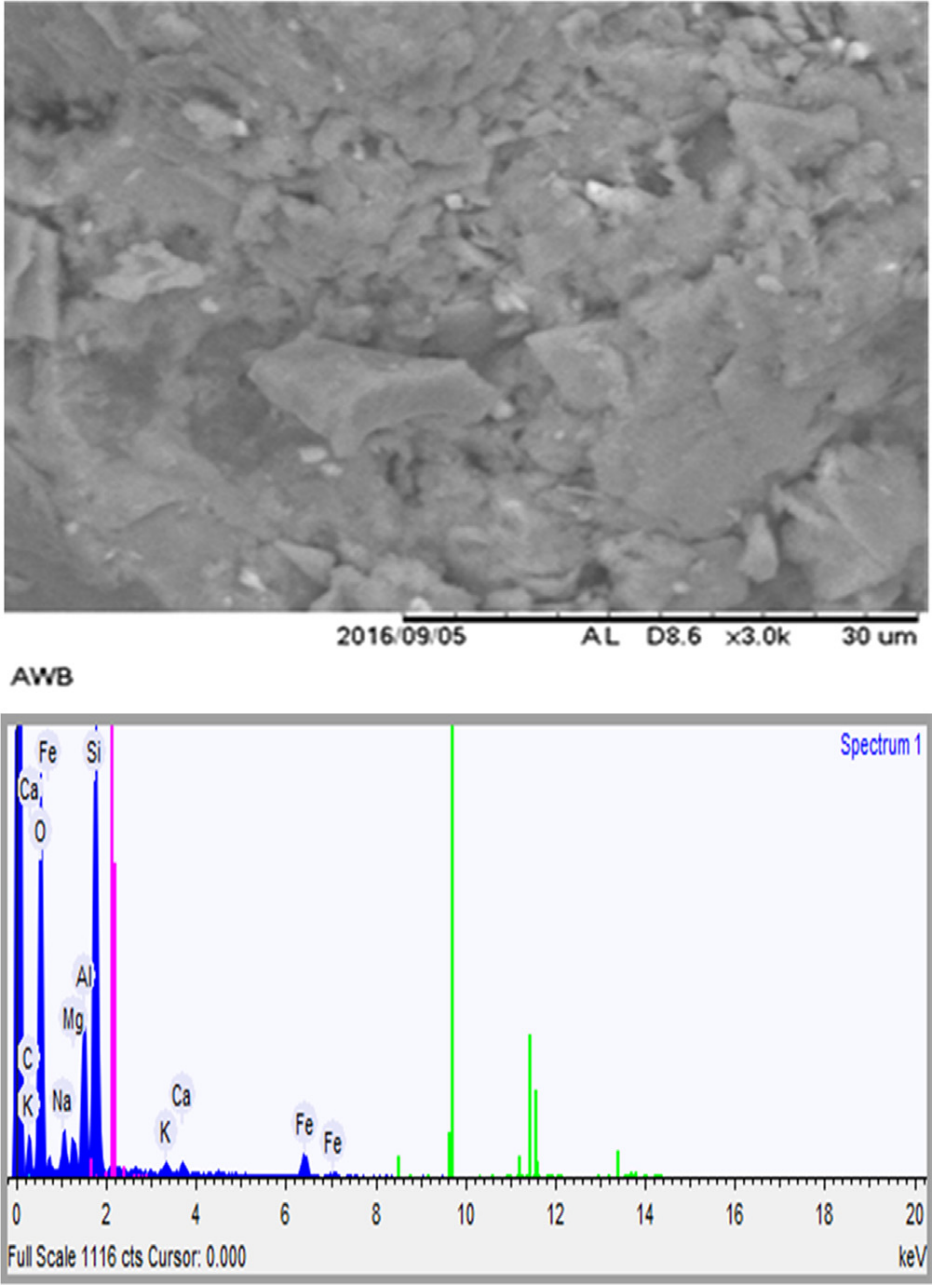
The results of SEM-EDX for the adsorbent from mixture of algae waste-bentonite before adsorption.

**Fig. 4:**
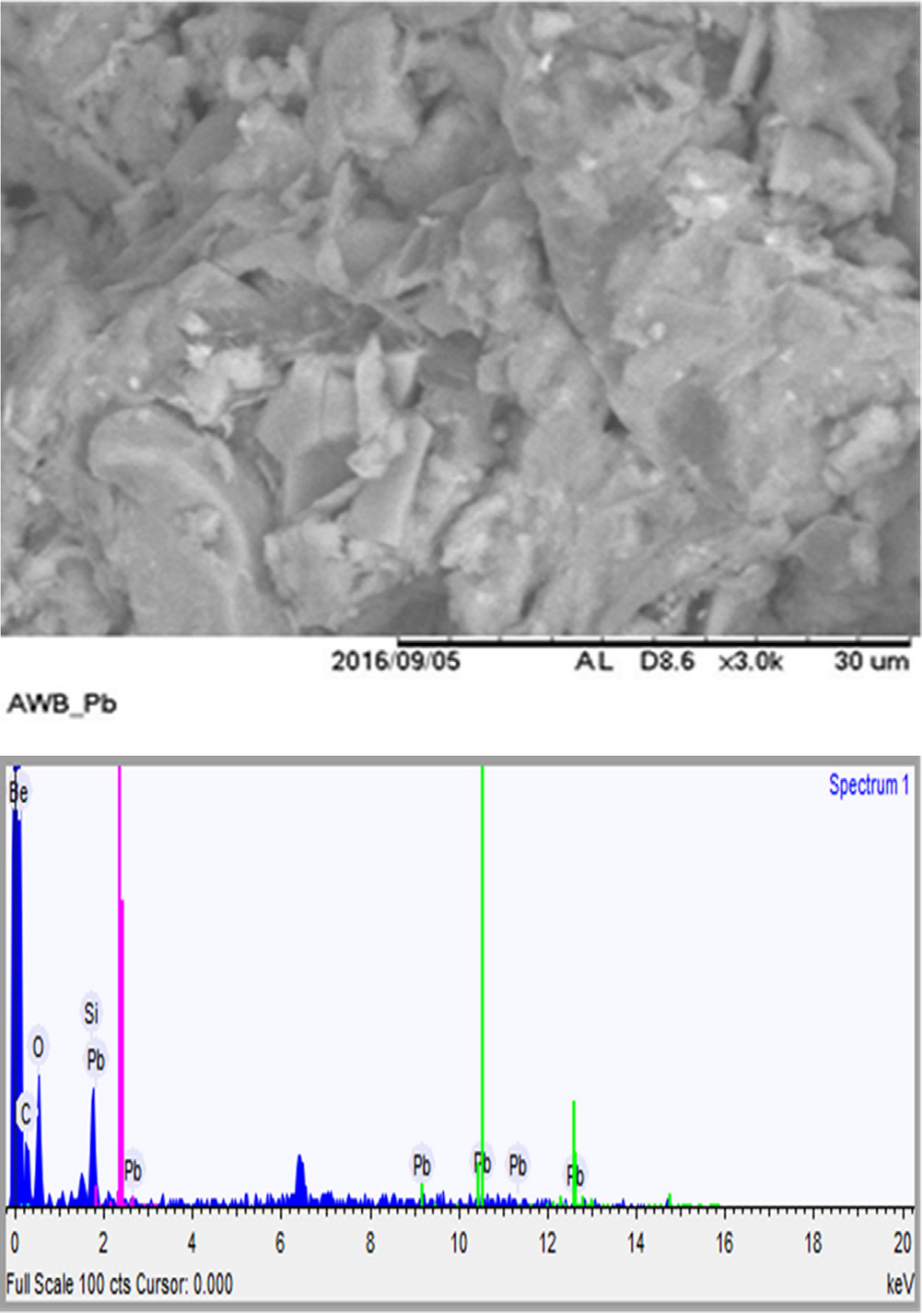
The results of SEM-EDX for the adsorbent from mixture of algae waste-bentonite after adsorption.

The FTIR for the adsorbent from mixture of algae waste-bentonite before and after adsorption at wave numbers from 400 to 4000 cm^−1^ were given in Fig. 1, Fig. 2. The SEM-EDX for the adsorbent from mixture of algae waste-bentonite before and after adsorption were given in Fig. 3, Fig. 4.

## 2 Experimental design, materials and methods

### 2.1 Materials

Red algae waste (Gracilaria sp.) was obtained from agar industry in Malang, East Java, Indonesia.

### 2.2 Preparation of adsorbent from mixture of algae waste-bentonite

The treatment given to algae waste was drying under sunlight for several days. The dried materials were then sieved to get the particle size of 100–200 mesh. Bentonite with the same particle size was then mixed with algae waste with the proportion of 2:1, and it was used as adsorbent. The lead solution was prepared by dissolving Pb(NO_3_)_2_ into demineralized water to get desired concentration.

### 2.3 Adsorption experiments

Adsorption of Pb^2+^ with the adsorbent of algae waste-bentonite was performed using batch adsorption technique [1], [2]. Adsorption experiments were carried out by adding 100 ml of 100 ppm lead solution placed in a 150 mL bottle and 0.5 g of adsorbent. The bottle was then placed on a shaker. After being shaken, the solution was then filtered and analyzed by atomic absorption spectrophotometer to determine metal concentration. First, the pH used in the present study was 2–7 to find out the effect of pH. Second, the adsorbent mass was 0.1–1.0 g to find out the effect of adsorbent dosage. The last, the contact time used were 10–240 min to find out the effect of contact time. All experiments were repeated three times.

### 2.4 Characterization of adsorbent from mixture of algae waste-bentonite

The characterization of adsorbent from mixture of algae waste-bentonite for before and after adsorption was carried out using scanning electron microscopy with energy dispersive X-ray (SEM-EDX) and fourier transform infrared (FTIR) [3]. The characterization of adsorbent from mixture of algae waste-bentonite was carried out using scanning electron microscopy with energy dispersive X-ray (SEM-EDX) which aimed to analyze and to find out the original micrographs and chemical composition on the surface of the adsorbent samples and fourier transform infrared (FTIR) which aimed to analyze and to find out the functional groups of adsorbent from mixture of algae waste-bentonite.

### 2.5 Data analysis

The efficiency of Pb^2+^ adsorption by adsorbent from mixture of algae waste-bentonite is calculated according to Eq. (1).

(1) $$\mathrm{Efficiency adsorption}=\frac{C_{o}\;-\;C_{e}}{C_{o}}\;\cdot \;100\%$$

where C_o_ is initial concentration (mg/L) and C_e_ is final concentration (mg/L).
